# Supplementary material for: Improved diagnosis of extrapulmonary tuberculosis in adults with and without HIV in Mbeya, Tanzania using the MPT64 antigen detection test
Source: PLOS Glob Public Health. 2022 Nov 29;2(11):e0001317. doi: 10.1371/journal.pgph.0001317 (PMC10021170; doi:10.1371/journal.pgph.0001317)
Supplement: Attachment — Submitted filename: Response to reviewers.docx [file pgph.0001317.s006.docx]

Response to reviewers

PGPH-D-22-01191
Improved diagnosis of extrapulmonary tuberculosis in HIV infected adults in Mbeya, Tanzania using the MPT64 antigen detection test
PLOS Global Public Health

Dear Sirs,	Oslo, 23.09.22

Thank You very much for Your comments on our article. I am confident that the article is much improved after the revisions You have suggested, and I hope that You agree. Thank You for Your time, effort, and advice.

Please find the responses to Your comments below.

Best regards,

Erlend Grønningen


Reviewer 1:
1. Please, change “HIV infected” as HIV-infected in the whole text –

Has been changed to PLWHIV in the text of the main body after comment from reviewer 2. In the tables it has been changed to “HIV-infected”.

2. RESULTS: is any possibility to have at least a figure with the data represented in a graph? A colored graph is welcome. This will help the reader to easily understand the message of the high accuracy of the MPT64 antigen detection test for the diagnosis of extra-pulmonary TB

Thank you for this important point. I have made a graph showing the correlation of positive results of mycobacterial culture/GeneXpert with MPT64 test results.

3. please, in the discussion, mention about the potential clinical implications of your findings for the diagnosis extra-pulmonary TB in the HIV-infected individuals vs HIV-uninfected in both, low and high resources countries.

Added sentence in the discussion:

Our results imply that the MPT64 test can diagnose EPTB with a high level of precision irrespective of HIV status in both high- and low resource settings

4. please, mention the use of IGRA in the context of extra-pulmonary TB stating the limits. You may also consider to mention Goletti D et al. Int J Infect Dis. 2022 Mar 5:S1201-9712(22)00126-6. doi: 10.1016/j.ijid.2022.02.047; Petruccioli E, J Infect. 2020 May;80(5):536-546. doi: 10.1016/j.jinf.2020.02.009.

Added following in discussion part, referencing Goletti article:

Serological test such as IFN-γ release assays lack accuracy to identify the progressors from infection to TB disease, and are not suitable to detect EPTB

Reviewer 2:

Major

The main issue is the results. I find it unnecessary to spend so much time discussing ZN staining results when it is already known that it performs poorly for EPTB. I suggest removing all results related to ZN throughout the results section. You even say in the discussion “ZN staining was of poor diagnostic value in this study”

I also find it strange that the authors spend so much time comparing the performance of culture and Xpert to the CRS and to each other. This paper is supposed to present findings related to MPT64, all these other results are distracting. I also don’t know what the sensitivity and specificity being reported is in comparison to for all these assays. Line 259: 28% sensitivity to what?

The word “correlation” to describe the relationships between assays in Table 4 is not the right word. These 2x2 comparisons are in fact describing the sensitivity/specificity relationships between assays.

My suggestion for the results is to keep the focus on MPT64. Remove all sens/spec/ppv/npv/accuracy for GeneXpert, culture, and ZN. Build 2x2 tables for all participants comparing it to 1) CRS; 2) Xpert; 3) culture. This will allow you to simultaneously compare the performance of MPT64 across different assays and draw conclusions on its performance. This means removing tables 2-4. These can be done for total pop, HIV-pos, HIV-neg. Supplementary can contain sample specific 2x2.

Response:

Thank you for these valuable inputs. They have enabled me to remove unnecessary information, structure the tables and figures and hopefully make the article more concise.

Tables have been re-done and information about ZN, GeneXpert and culture will mainly be moved to the supplementary. However, one table stating the sensitivity, specifivity, PPV, NPV and accuracy for all tests in different groups will stay (labelled table 3). This as the objectives of the study was to validate the MPT64 test in PLWHIV against currently used tests in addition to against the CRS.

The tests are compared against the CRS, meaning that a sensitivity of 28% is compared to the CRS. A sentence has been added for each test to specify this.

Table 2 is a new table that now shows the performance of MPT64 across different specimens in PLWHIV/HIV negatives and includes the p-value calculations for the difference in performance relating to serostatus. This table was previously labelled table 2, but all information about other diagnostics tests have been removed. This information is now found in supplementary table 1 (which equals the old table 2).

The results for MPT64 is now mentioned first in the text. Information about ZN is kept in the results section, but removed from the discussion.

Table 3 is kept as it was to show the performance of other test to validate the MPT64 not only against the CRS in HIV-/HIV+, but also against other tests.

Table 4 has been transformed into a figure (figure number 3) that shows the correlation of positive mycobacterial culture. Figure introduced on request of previous reviewer. The previous table 4 information is now found in the text.

For tables 5 and 6, remove ZN, culture, Xpert. Shouldn’t table 5 be PLWHIV and table 6 HIV negative instead of TB positive and TB negative? The objective of the paper is to compare PLWHIV with HIV negative so combining these two groups doesn’t answer the research question. Either way, would consider merging these two tables so that the two groups (whatever they are) are side by side for easy comparison.

Response:

Agreed. This greatly improves the table and reduces the disturbance from all the findings about the other tests and their correlation to FNAC and cytology. This information will be found in the supplementary. Labelled supplementary table 2 and 3.

The tables previously called table 5 and 6 are now called table 4 and only contains information about the MPT64 test and correlation to FNAC and cytology findings.

To account for changes in the tables, the results part has been greatly reduced and reference to information about the performance of ZN, GeneXpert and mycobacterial culture will be moved to tables in the supplementary.

Discussions part has also been changed to reduce information about other tests, in line with comments below.

Minor

• Please use people living with HIV (PLWHIV) throughout instead of “HIV infected”.

Has been implemented throughout the text.

• Line 1: Is this study truly focused on PLWHIV? The title makes it seem so, however more than half the study population were not living with HIV. I think the title needs to be clarified that this is a comparison the performance between the two populations.

Answer:

Yes. It is about PLWHIV, but a comparison is needed as you mentioned. Have changed the title to account for this:

Improved diagnosis of extrapulmonary tuberculosis in adults with and without HIV in Mbeya, Tanzania using the MPT64 antigen detection test

• Line 30: Tanzania, not an “African setting”

Thanks for the comment. Changed to Tanzanian.

• Line 36: general information about what the CRS was could be useful (test based, clinical based etc)

Added clinical suspicion, mycobacterial culture, cytological and radiological findings.

• Line 44-45: the long list of percentages is very difficult to read. Please put these immediately next to the statistic it represents to make it more clear “sensitivity (91% vs 78%)”

Agreed. Changes introduced as proposed.

• Line 85-88: references are missing for these sentences

Reference introduced:

Purohit M, Mustafa T. Laboratory Diagnosis of Extra-pulmonary Tuberculosis (EPTB) in Resource-constrained Setting: State of the Art, Challenges and the Need. J Clin Diagn Res. 2015;9(4):EE01-6.

• Line 83-94: reference missing for AFB staining

Added references:

Purohit MR, Mustafa T, Wiker HG, Sviland L. Rapid diagnosis of tuberculosis in aspirate, effusions, and cerebrospinal fluid by immunocytochemical detection of Mycobacterium tuberculosis complex specific antigen MPT64. Diagn Cytopathol. 2012;40(9):782-91.

Perkins MD, Roscigno G, Zumla A. Progress towards improved tuberculosis diagnostics for developing countries. Lancet. 2006;367(9514):942-3.

• Line 97-102: is the info on LAM and ADA tests necessary? You never use these assays so it just feels like it extends the intro unnecessarily

Agreed. Removed from text. Introduces noise in the article as these tests are not examined in this study.

• Line 127-128: Is there a reference for any published papers that used this cohort?

This is the first publication on the adult cohort. Introduced reference to the pediatric cohort.

• Line 142: Self-reported HIV status could introduce some sort of bias, which could under estimate the number of people who are truly positive, which could be related to social desirability or recall. Might be worth mentioning in the limitations and suggesting that a more thorough approach would be to test participants for HIV at enrollment

Agreed. The study participants were also asked to disclose which medications they were using (ARVs?), and this was used a checkpoint. As you suggest, testing would have been better. Introduced the following in the discussion:

Patients were not tested for HIV as part of the study, which potentially could have reduced the number of people who were truly HIV-infected.

• Line 150: you are missing a clear definition of the CRS in this section – what assays/components form the CRS. Even if it is presented in the figure I think it should be repeatd here. You should also explicitly state that the definition for each category is in figure 1 if you do not wish to define it explicitly in the text.

Thanks for the comment. Have added information about which assays/components that formed the CRS. In addition, there is already a sentence in the text that tells that the definition of each figure is found in Figure 1. The following is added:

The CRS included clinical suspicion, mycobacterial culture, concomitant confirmed PTB, response to anti-TB treatment, radiological findings, cytology findings compatible with EPTB in effusions and morphological features on FNAC

• Line 202: You talk of “agreement” as a form of quality control. How were these 20 slides selected? Perhaps you can quantify the level of agreement instead of reporting number of concordant/discordant pairs. Also, if LS labelled 7 inconclusive and the other examiner provided a result these are also part of the discordant pair, representing an agreement of 55%

Thanks for the requested clarification. Have done the analysis over again and updated numbers for more precision. Agreement is here seen as a form of quality control as Mbeya Zonal Referral Hospital did not perform immunostaining before our study was conducted. LS had experience in assessing a variety of immunostainings and is a very experienced pathologist. Agreement between a more junior (WM and LT) pathologist in the evaluation of immunistainings and a senior (LS) was seen as a form of quality control.

Text changed to:

To assess the quality of the reading of the MPT64 test an independent reading of 20 randomly selected immunostained slides was performed by a pathologist from a Norwegian university hospital (LS). Agreement was found on 12/20 (60% ) MPT64 slides (55% positive, 5% inconclusive). 8/20 (40%) had a discordant result. Among the discordant cases 6 (30%) were labelled as inconclusive by LS, 1 (5%) positive by the local pathologist and negative by LS and 1 (5%) positive by LS and inconclusive by the local pathologist.

• Table 1: Specify that “self reported previous TB” is treatment >1 year prior to enrollment. Need to include text under the table defining all the abbreviations like IPD, OPD, TB etc.

IPD/OPD changed to Department. All other abbrevations are introduced earlier in the text.

Self reported previous TB treatment changed to: **Self-reported previous TB treatment (>1y prior to enrollment)**

• The discussion section is unusually long. This needs to be greatly reduced to make it succinct.

Discussion has been reduced. Removing much of the discussion point on the other test.
